# Accuracy of the references in JIAPS

**DOI:** 10.4103/0971-9261.43826

**Published:** 2008

**Authors:** Anup Mohta

**Affiliations:** Department of Pediatric Surgery, Chacha Nehru Bal Chikitslaya and Maulana Azad Medical College, Delhi - 110 031, India

Sir,

References of a biomedical journal form an important component of a scientific communication. Accurate references add to the credibility of the publication, authors and the journal. This study was conducted to find out the accuracy of references in the Journal of Indian Association of Paediatric Surgeons (JIAPS).

All the 114 references in the articles published in the January-March 2008 issue of JIAPS were listed. An attempt was made to verify the references from the original articles or from the internet sources available. A total of 106 (91%) references could be checked. References from the text books and other publications (n = 8) could not be verified, which included one reference from a journal due to nonavailability.

A total of 29 references were found to have errors (27.5%). Major errors included wrong first author name (n = 7), wrong journal name (n = 2), wrong year (n = 1), wrong volume number (n = 1) and wrong first page number (n = 2). Minor errors included wrong coauthors’ names (n = 22), wrong number of coauthors (n = 5), short title (n = 1), wrong journal abbreviation (n = 1) and wrong last page number (n = 2). The most common error was found to be in the names of authors in different forms. Two internet references were wrongly cited and one of these could not be traced.

References in a scientific publication are a source of information for the readers. It is very important for the references to be correct as incorrect references frustrate the reader while searching for related articles.[1] Despite editorial instructions for checking the references accurately before a manuscript is submitted for publication and availability of various electronic resources, this continues to be a major problem in almost all the specialty journals. Mohta and Mohta[1] found an error rate of 80% in a surgical speciality journal from India while an earlier study by Evans et al,[2] found an overall error rate of 48%. The error rate in different pediatric journals has been found to vary from 29 to 39%.[3,4] Although the number of internet citations in scientific communications is still very low,[5] these have been found to become inaccessible very quickly.

The primary responsibility of checking the references lies with the authors, but the editorial board and the reviewers also need to be vigilant by randomly checking the references. JIAPS needs to be complimented for a low error rate as compared with other journals, but this needs to be further brought down. This can only be done by more careful citations provided by the authors after checking primarily from the original articles. Cross references need to be thoroughly verified before citation. Internet citations need to be carefully cited as per guidelines. JIAPS should include a sample of the format in the common examples given in the "Instructions to Authors," as their use is likely to increase in the near future.
